# Reduction of Dislocations of the Femur by Manipulation

**Published:** 1858-06

**Authors:** Frank Hastings Hamilton

**Affiliations:** Buffalo, N. Y.


### BUFFALO MEDICAL JOURNAL

AND

### MONTHLY REVIEW.

**VOL. 14.** JUNE, 1858. **NO. 1.**

### ORIGINAL COMMUNICATIONS.

ART. I.—*Reduction of Dislocations of the Femur by Manipulation. Fragments of the Literature of this Method.* By FRANK HASTINGS HAMILTON, M. D., Buffalo, N. Y.

(Continued from Page 589.)

The following is from a letter written by Dr. Jno. T. Plummer, a distinguished quaker practitioner, residing in Richmond, Indiana, to Dr. Hill, editor of the Medical Counsellor:

"In looking over the last number of the Counsellor, I was surprised to read in a communication copied from the New York Journal of Medicine, that a certain 'vastly improved method' of reducing luxations 'originated with Dr. Reid.' The writer calls it 'Dr. Reid's Method of Manipulation,' etc. * * * * From the date and language of the communication it would appear that this mode of reducing dislocations of the head of the femur was of quite late origin. I commenced practice in the year 1828, and from that day to this, I have used no other mode of restoring the bone to to its place than that described by the correspondent of the N. Y. Jour. of Medicine.

"One of the earliest cases that fell to my lot occurred in a young able-bodied man under circumstances which made it very desirable to him that others should not be present at the reduction. The dislocation was upon the dorsum ilii. By engaging the attention of the patient in another direction while I manipulated his leg, I readily reduced it without assistance.

"But I must not forget to state that my object is not to claim priority of discovery or practice in this point of surgery, but simply to express my surprise that this method of reduction should not have been more extensively known than it would appear to be. I cannot positively say, but I am willing and inclined to believe that I obtained the hint on this subject from Dr. Nathan Smith, then of the Medical College of New Haven, formerly of Dartmouth. I know he was remarkable for simplifying as much as possible all the processes of surgery. Complex surgical machinery he had no relish for."—*Medical Counsellor, Columbus, Ohio, Feb.* 2, 1856, *p.* 117.

I shall make only one more quotation, intended to illustrate Dr. Nathan Smith's connection with the history of reduction of the hip-joint by manipulation:

"But one case of luxation of the os femoris has fallen to my care, which I was able to reduce without a recourse to forcible extension. This occurred about twenty years since, when the late Prof. Nathan Smith's account of the reduction of a similar dislocation was fresh in my recollection. It was a luxation upwards on the dorsum ilii. The method by which Dr. Smith ultimately succeeded in his case, after fruitless attempts by extension, was the one by which I had determined to attempt reduction; but that I might be prepared for a failure, towels and bands were applied as usual, with assistants ready to make any extension which might be found necessary. The patient was permitted to lie on his back on the bed where I found him, the knee of the luxated limb turned in and over the other. I raised the knee in the direction it inclined to take, which was towards the breast of the opposite side, till the descent of the head of the bone gave an inclination of the knee outwards, when I made use of the leg, being at right angle with the thigh, as a lever to rotate the latter and turn the head of it inwards. It then readily returned to its socket, with an audible snap. During this operation the two assistants who had been placed to make the lateral extension and counter-extension, if ultimately required, were directed to draw moderately at their towels. How much of the success of the operation is to be imputed to their extension, and the rotation of the thigh by the leg, I am unable to determine; but as Dr. Smith succeeded without the aid of either, and as the head of the femur seemed to descend by an easy and natural process, I am inclined to believe that all that is necessary in such cases, is to elevate the knee, when the ilium, the muscles attached to it, and perhaps the ligaments, become the natural fulcrum, over which the thigh, as a lever, acts to bring the head down and inwards into the socket. Indeed it would be in vain to attempt to bring the head of the bone into its place, until it had presented itself to the socket, by any power whatever, as was fully illustrated in the case recorded in the French Lancet and elsewhere. In this case of dislocation of the thigh, during seven months and a-half, after powerful and continued extension for several days, and 'when the head of the femur was brought down to a level with the acetabulum, the extending force was suspended, and the two assistants having bent the leg on the thigh, were directed to rotate the latter from without inwards; during this manœuvre the femur was broke across the lower third.'"—*Boston Med. and Surg. Jour., vol.* xxii, *p.* 249, *May*, 1840. *Paper by Luke Howe, of Boston, Mass.*

TABLE, COMPRISING 64 CASES OF DISLOCATION OF THE HIP, REDUCED BY MANIPULATION. COLLECTED BY LUCIEN DAMAINVILLE, STUDENT OF MEDICINE, MARCH, 1858.

| No. | Age. | Sex. | Form of Dislocation. | Time since it occurr'd | What means employed before. | When manipulation employed. | What means us'd to diminish resistence of muscles. | Accidents, Results, &c. | Operator. | References. |
|---|---|---|---|---|---|---|---|---|---|---|
| 1 | Adult. | M. | Fora'n ovale. | Few h'rs | Pulleys. | Sept'ber 1772. | Not any. | Reduced in first att'pt at work in 3 weeks and without the least stiffness in the joint. | Thos. Anderson, of Leith, Scotland. | "Medical Commentaries," Edinb'g, 1776, vol. ii, p. 261. |
| 2 | 8 yrs. | M. | Dorsum ilii. | 19 days. | Not any. | | Not any. | Reduced in first att'pt. Could walk in three weeks, but compl'd of stiffness in joint for a week or two more. | " | " |
| 3 | 40 yrs. | M. | Isch'tic notch | 5 hours. | Pulleys. Four attempts continued 1 hour. | Jan. 7th, 1811. | Vena sec | Reduced accidentally. | Philip Syng Physick, of Philadelphia. | Memoirs of Nathan B. Smith, p. 172. Also Dorsey's Surgery, v. i, page 242. Also Trans. of N. Y State Med. Soc., 1856, p. 167. |
| 4 | | | Dorsum ilii. | | "Had pulled in every other direction but the right one." | Prior to 1824. | | Reduced easily. | Nathan R. Smith, of New Haven, Ct. | "Charles Lowell *vs.* John. Faxon," &c. Trial for malpractice. See also this Journ., Feb. 1858, p. 515; also Smith's Memoirs, *op. cit.* |

| | | | | | | | | | | |
|---|---|---|---|---|---|---|---|---|---|---|
| 5 | | | | | | Prior to 1824. | | Reduced easily, by the same method as in case 4. | —— of Mass. | Smith's Memoirs, *op. cit.*, p. 177. |
| 6 | | | | Few h'rs | Pulleys. | " 1831. | | Reduced easily. | By an empiric. | " p. 170. |
| 7 | 13 yrs. | M. | Dorsum ilii. | Few h'rs | Not any. | Jan. 23d, 1854. | Ether. | | Sam'l Shumway, of Essex Co., N. Y. | Transactions of N. Y. State Med. Society, 1854, p. 55. |
| 8 | Adult. | M. | Pubes, comp. dislocation. | 12 or 14 hours. | Powerful manual extension, &c. | Prior to 1842. | Opium & vena sect'n. | Reduced easily in 1st attempt. Died in 3 weeks with extensive suppuration about the joint. | William Ingalls, of Boston, Mass. | B. Cooper's ed. of A. Cooper, on Dislocations, &c., p. 586. Also this Jour. Mar. 1858. |
| 9 | " | F. | Dorsum ilii. | 4 days. | Not any. | Spring of 1844. | Vena sec. & ant. | 1st attempt. | W. W. Reid, of Rochester, N. Y. | Buff. Med. Journ., vol. vii, p. 137, Aug. 1851 |
| 10 | 38 yrs. | F. | " | 1 hour. | Not any. | July 31, 1849. | Morph. | 2d attempt. Reduc'd in two minutes. | " | " p. 139. |
| 11 | 52 yrs. | M. | " | 8 hours. | Not any. | Dec. 2d, 1849. | Nothing. | 1st attempt, 2 or 3 minutes occupied. | " | " p. 140. |
| 12 | Adult. | M. | " | Few h'rs | Not any. | 1851. | Chloroform & ether. | 1st attempt, 30 sec'ds. | A. Barnard, Adrian, Michigan. | New York Journal of Med., Nov., 1851, p. 409. |
| 13 | 4 yrs. | F. | " | Few h'rs | Not any. | Feb. 14, 1851. | Nothing. | 1st attempt successful, rapid and complete recovery. | J. M. Litten, of Austin, Texas. | " March, 1852, p. 259. |
| 14 | 31 yrs. | M. | " | 13 days. | "Repeated att'pts even by the pulleys." | | | | Mahr. | " March, 1852, p. 270. |

| | | | | | | | | | | |
|---|---|---|---|---|---|---|---|---|---|---|
| 15 | | | | | Not known. | | Nothing. | Accidental, while 'making some preliminary movements.' | John C. Cheeseman. of New York. | New York Journal of Medicine, Jan. 1855. *Markoe's paper.* |
| 16 | Adult. | M. | Dorsum ilii. | Few h'rs. | Jarvis' adjuster, with ether; adjuster bioke. | Nov. 30, 1852. | Ether. | 2d attempt. Perfect result. | Gurdon Buck, of N. York. | " |
| 17 | 25 yrs. | M. | Dorsum ilii. | Few h'rs. | | Dec, 8th, 1852. | Nothing. | 2d attempt successful. Perfect result. | Halsted, of N. Y. | " |
| 18 | 37 yrs. | M. | Dorsum ilii. | 3 m. | A surgeon had tried, but failed. It was suspected that the acetabulum was brok'n; whether by the accident or not, is not stated. | | Ether. | 1st attempt failed; 2d succeeded, but dislocation returned & could not afterwards be reduced. Shortened, but pretty useful limb. | Jno. Watson, of N. York. | " |
| 19 | 21 yrs. | M. | Isch'tic notch | Few h'rs. | Not any. | May 22, 1853. | Ether. | 1st attempt; thrown on obdurator foramen. After being moved backwards & forwards between these points several times, it was finally carried from the foramen ovale into the socket by the common mode of extension and lifting. An abscess followed & probably morbus coxarius. | Alfred Post, of N.Y. | " |

| | | | | | | | | | | |
|---|---|---|---|---|---|---|---|---|---|---|
| 20 | 8 yrs. | M. | Dorsum ilii, with comp. fracture of same thigh | Few h'rs | Not any. | June 29, 1853. | Nothing. | Did well. | House Surgeon of N. Y. Hospital. | New York Journal of Medicine, Jan. 1855. *Markoe paper.* |
| 21 | 28 yrs. | M. | Dorsum ilii. | ½ an hr. | Not any. | | Ether. | Two first trials bro't it towards the foramen ovale. 3d trial, with only slight abduction successful. | T. M. Markoe, of N. York. | " |
| 22 | Adult. | M. | Foramen ov. | 1 month. | Been regarded as a sprain by his surgeon. | Dec. 12, 1853. | Ether. | 1st, carried upon the dorsum ilii; 2d, back again; 3d, upon dorsum; 4th, back again; several more unsuccessful att'pts were made, and finally it was reduc'd by pulleys. A good deal of swelling and pain followed, in the joint; but he was discharged cured, on Jan. 13th. | Halsted, of N. Y. | " |
| 23 | 22 yrs. | M. | Foramen ov. | Not long | Extens'n & lifting by usual mode; without pulleys | April 22, 1854. | Ether. | 3d effort successful. Recovery rapid. | Gurdon Buck, of N. York. | " |
| 24 | 38 yrs. | M. | Foramen ov. | 14 days. | 1st surgeon tho't it a contusion. | May 23, 1854. | Ether. | 1st attempt failed; 2d attempt threw head upon ischiatic notch; 3d attempt after restoring head to foramen ovale, successful. Rapid recov'y. | T. M. Markoe, of N. York. | " |

| | | | | | | | | | | |
|---|---|---|---|---|---|---|---|---|---|---|
| 25 | Adult. | M. | Isch'tic notch | V'y soon after. | Not any. | | Ether. | | Dewitt C. Peters, U. S. A. | New York Journal of Medicine, Jan. 1855. *Markoe paper.* |
| 26 | 40 yrs. | M. | " | " | Not any. | Jan. 11, 1853. | Chloroform. | First attempt carried head of bone upon foramen ovale. 2d, successful. Recovery rapid. | Willard Parker, of New York. | " |
| 27 | 44 yrs. | F. | Dorsum ilii. | Few hrs. | Not any. | Nov. 1st, 1854. | Chloroform. | | " | " |
| 28 | 37 yrs. | M. | Dorsum ilii, with fracture same leg below the knee. | Soon after. | Not any. | Nov. 22, 1854. | Ether. | 1st attempt failed; 2d attempt failed; 3d, pulleys failed; 4th, Jarvis' adjuster failed; 5th, manipulation failed; 6th, do. failed; 7th, manipulation succeeded. | John Watson, of N. York. | " |
| 29 | 42 yrs. | M. | Dorsum ilii. | 7 weeks. | Not any. | Oct. 25, 1854. | Ether. | 1st attempt head went upon foramen ovale. Numerous unsuccessful trials followed. The head was then placed on foramen ovale and Sir A. Cooper's method tried, breaking the cervix femoris. Reduct'n never accomplished. Fair recovery. | T. M. Markoe, of N. York. | " |
| 30 | 21 yrs. | M. | Isch'tic notch | 2 hours. | Not any. | March 23 | Nothing. | 1st attempt failed; 2d | Frank H. Hamilton, | Buff. Med. Journ., vol. |

| | | | | | | | | | | |
|---|---|---|---|---|---|---|---|---|---|---|
| | | | | | | 1855. | | attempt successful. Walked in 11 days. Slight soreness and pain continued for a few months. | of Buffalo, N. Y. | xi, p. 210. Also N. Y. Jour. Med., Nov., 1855, and Am. Jour. Med. Sci., Oct. 1855 |
| 31 | Adult. | F. | Dorsum ilii. | Soon after. | Not any. | Oct. 7th, 1855. | Nothing. | 1st attempt successful. | E. J. Fountain, of Davenport, Iowa. | N. Y. Journ. of Med., Jan., 1856, p. 69. |
| 32 | " | M. | Pubes. | " | Not any. | June, 1854. | Chloroform. | 1st attempt successful. | " | " |
| 33 | " | M. | " | " | Not any. | Oct. 31, 1855. | Nothing. | 1st attempt successful. | " | " |
| 34 | | | | 5 weeks. | Pulleys. | | | | Cock, of London. | N. Y. Journ. of Med., Jan'y, 1856, p. 143, from Lancet, July 7, 1855. |
| 35 | | | | | " | | | | " | " |
| 36 | | | Isch'tic notch | | | | | | Birkett, of London. | From Lancet of Aug. 4th, 1855. |
| 37 | | | Foramen ov. | | | | | | " | " |
| 38 | | | " | | | | | | " | " |
| 39 | 28 yrs. | M. | Dorsum ilii. | 6 days. | Nothing. | Jan. 7th, 1858. | Chloroform. | 7th trial successful; meantime the head was carried upon foramen thyroid and back repeatedly. Recovery rapid. | Frank H. Hamilton, of Buffalo, N. Y. | Buffalo Med. Journal, April, 1858, p. 682. |
| 40 | 28 yrs. | M. | Isch'tic notch | Soon after. | Not any. | Feb. 23, 1856. | Ether. | | James R. Wood, of New York. | N. Y. Jour. Med., May, 1856, p. 399. |

| | | | | | | | | | | |
|---|---|---|---|---|---|---|---|---|---|---|
| 41 | 8 yrs. | M. | Isch'tic notch | 2 days. | | October, 1857. | Chloroform. | Succeeded in less than ½ a minute. Patient "has done well." | C. E. Isaacs, Brooklyn, N. Y. | N. Y. Jour. Med., Nov., 1857, p. 418. |
| 42 | 7 yrs. | M. | Dorsum ilii. | 5 hours. | Not any. | 1855. | Nothing. | 1st attempt failed; 2d attempt successful. Both attempts did did not occupy more than one minute. Recovered perfectly in a few days. | ——— of Michigan. | Peninsular Journal of Med., July, 1855, p. 35. |
| 43 | | | " | 6 mon's. | | Mar. 22, 1856. | Chloroform. | No "unpleasant effects" followed. | Blackman, of Cincinnati. | Ohio Med. and Surg. Jour., vol. viii, p. 522 from West'n Lancet. |
| 44 | 23 yrs. | M. | " | Several hours. | Manual traction for 1 hour, under influence of chlorof'm, subsequently pulleys. | June 1855. | " | 1st attempt successful. | E. Cock, of London. | Braithwaite, Jan. 1856, from Med. Times & Gaz., June 30, 1855, p. 644. |
| 45 | Adult. | F. | Foramen ov. | 8 hours. | Extension under the influence of chloroform. | July 28, 1855. | " | 1st attempt successful. | Welch, of London. | Braithwaite, July, 1856 from Med. Times & Gaz., April 12, 1856, p. 362. |
| 46 | 2 y. & 1 m. | M. | Isch'tic notch | 1 day. | Not any. | Mar. 28, 1852. | Not any. | 1st attempt successful. Was soon well. | J. H. Beech, of Coldwater, Mich. | Buffalo Med. Jour. vol. viii, p. 71. |
| 47 | | | " | Recent. | " | 1852. | Ether. | Immediately reduced. | Parkman, of Boston. | Amer. Jour. Med. Sci., Jan., 1853, p. 79. |
| 48 | 7 yrs. | M. | Dorsum ilii. | 3 hours. | " | 1857. | Not any. | 1st attempt successful. Occupied few sec'ds | Chas. H. Baker, of Alexander, N. Y. | Buff. Med. Journ., vol. xii, p. 624. |

| | | | | | | | | | | |
|---|---|---|---|---|---|---|---|---|---|---|
| 49 | 7 yrs. | M. | Dorsum ilii. | | | 1857. | Ether & Chloroform. | Reduced in 30 sec'ds. | T. G. McElbright, of Ohio. | Amer. Journ. of Med. Sci., July, 1857, p. 292, from Western Lancet, April, 1857. |
| 50 | 40 yrs. | M. | Isch'tic notch | Soon after. | Not any. | 1857. | Chloroform. | "Leg very much everted." 3d att-pt successful. Recovery rapid. | G. R. Henry, Burlington, Iowa. | North Amer. Medico-Chirurg. Rev., vol. i, p. 933. |
| 51 | 6 yrs. | M. | Dorsum ilii. | Few hrs. | Not at first recognized. | Aug. 29, 1855. | " | 1st attempt successful. | M. H. Stapleton, of Ireland. | Am. Jour. Med. Sci., April, 1856, from Dublin Med. Press, Feb. 13, 1856. |
| 52 | 44 yrs. | M. | Foramen ov. | 3 weeks. | First surgeon supposed it to be a sprain. | July 25, 1856. | " | Succeeded in 10 min. | Wormald, of England. | " Oct., 1856, p. 513, from Med. Times & Gaz., Aug., 1856. |
| 53 | 22 yrs. | M. | Isch'tic notch | 6 weeks. | First tri'd pulleys, which drew it from Dorsum ilii into the notch. | | | | " | " |
| 54 | 16 yrs. | M. | Dorsum ilii. | 6 mos. | Manual extension. | | | 1st attempt successful. Reluxated and reduced three times. | Dupierris, of Havana, Cuba. | Iowa Med. Jour., May, 1857, from North Amer. Med. Rev. |
| 55 | 34 yrs. | M. | Foramen ov. | 3 hours. | Not any. | July 23 1855. | Ether. | 1st attempt successful. Perfectly well in 8 days. | W. H. Van Burrey, of New York. | N. York Med. Times, Jan., 1856, p. 126. |
| 56 | 42 yrs. | M. | Isch'tic notch | Few hrs. | " | July 28, 1855. | " | 1st attempt successful. Well in 12 days. | " | " |
| 57 | 21 yrs. | M. | Isch'tic notch | Few hrs. | Not any. | Aug. 23, | " | Tried seven times and | " | " |

| | | | | | | | | | | |
|---|---|---|---|---|---|---|---|---|---|---|
| | | | with fracture of opposite leg. | | | 1855. | | failed; 2d at'pt with extension succeed'd. Patient did well. | | |
| 58 | 15 yrs. | M. | Foramen ov. | Few hrs. | Not any. | Juue 17, 1857. | Chloroform. | 1st attempt successful. | R. L. Brodie, U. S. A. | Memphis Med. Rec., Sept., 1857, p. 90. |
| 59 | Adult. | M. | Not stated. | Few seconds. | " | Feb. 20, 1851. | Nothiug. | Dr. Swain was himself the subject of the dislocation, and he reduced it himself, by simply flexing thigh | Marcus Swain, of Essex, Vt. | Buffalo Med. Jour. vol. vii, p. 780, June, 1851. |
| 60 | | M. | Dorsum ilii. | | | 1820. | Nothing. | 1st effort successful. | Luke Howe, of Boston, Mass. | Boston Med. and Surg. Journ., vol. xxii, p. 249, May 27, 1840. |
| 61 | | | " | 4 days. | Jarvis' adjuster & Reid's mothod. | Febru'ry 1859. | Nothing. | Reid's method was tried several times but only carried the head of the bone from the dorsum to the ischiatic notch and back. Finally succeeded by his own method. Flexion and adduction, with some extens'n. | Moses Gunn, of Detroit, Mich. | Peninsular Journal of Med., July, 1855, p. 27. |
| 62 | | | | 7½ mos. | Extension for several days. | Prior to 1840. | | Flexion and rotation. During this manœuvre, femur was broken through its lower third. | | Boston Med. and Surg. Jour., vol. xxii, p. 249, (1840,) from "French Lancet." |
| 63 | Young man. | M. | " | | | | | | Jno. T. Plummer, of Richmond, Ind. | Medical Counsellor, Feb. 2, 1856. |
| 64 | Adult. | M. | Dorsum ilii. | 24 hours. | Not any. | | Nothing. | Manipulation. 1st attempt successful. | J. W. Dickey, Glasgow, Ky. | L'ville Rev., vol. i, No. 1, p. 103, May, 1856. |

*Resume.* Finally, our researches, by no means complete, have brought to light the following facts:

Richard Wiseman, so early as the year 1676, recommended, in case of children, where the thigh was luxated upon the dorsum ilii to adduct the limb, then flex it upon the belly, and thus reduce the bone.

Richard Boulton, in 1713, and Daniel Turner, in 1742, have repeated the same advice. The latter recommended also the application of a similar plan *in all cases*, before resorting to the pulleys; he has, moreover, reported one case at length in which he succeeded, by manual extension and manipulation, but without the aid of mechanical appliances; and he declared that he had reduced two others by the same method.

Heister, in 1768, thought also of reducing these dislocations by manipulation alone.

Sept. 1776, Thomas Anderson, of Leith, reported in the Edinburgh Med. Commentaries, two cases which he had reduced by manipulation, one being a dislocation upon the dorsum ilii, and one upon the foramen ovale.

Klug, in 1823; Wattman, in 1826; Rust, in 1828; Colombat, in 1830; Fishcher and Mahr, in 1849; published descriptions of their several modes of procedure; all of which were by manipulation, and most of which were essentially or precisely like that which is now generally practiced.

Our researches have also brought to light the following facts illustrative of the connection of American surgeons wish this procedure.

Dr. Philip Syng Physick accidentally reduced a dislocation of the hip by manipulation, in 1811.

Dr. Nathan Shumway, of Essex Co., N. Y., Dr. Luke Howe, of Boston, and many others affirm that Dr. Nathan Smith, of New Haven, taught and demonstrated upon the skeleton this mode of reduction of dislocated hips, in his lectures at Yale College, as early as 1816.

In 1824, Dr. Smith, in a written deposition, declared that he had once reduced a dislocation upon the dorsum ilii by carrying the knee towards the patient's face, and without pulleys.

In the year 1331, Dr. Nathan R. Smith, of Baltimore, published a full account of the views and practice of his father, Nathan Smith, in an octavo volume. His description of his mode of reducing dislocated hips, and his explanation of the theory of the process is clear and elaborate, occupying twenty pages or more. The whole is illustrated by wood cuts.

In May, 1840, Dr. Luke Howe called attention again to Dr. Smith's mode, in the Boston Medical Journal, and reported a case reduced by himself.

In the English edition of Sir Astley Cooper on Dislocations and Fractures, edited by Bransby Cooper, and issued in 1842, is an account of a compound dislocation of the hip reduced by manipulation. The same is seen also in the American edition of the same book published in 1852.

On the 8th of May, 1850, W. W. Reid, of Rochester, N. Y., read before the Monroe Co. Med. Society, a paper entitled "Dislocation of the Femur on the Dorsum Ilii, reducible without pulleys, or any other mechanical power," which paper, containing the details of three cases in which he had accomplished reduction by manipulation, was published in this Journal for August, 1851.

If my readers have followed carefully the descriptions which I have given of the modes of manipulation adopted by the various surgeons during the last two hundred years, they have seen a remarkable similarity in their procedures, especially when they have been speaking of dislocations upon the dorsum ilii, and such as to induce a belief that the modes were nearly or quite identical; and yet they must have noticed also constantly such slight differences in the descriptions, or in the terms employed, as to render it apparent that only in very few instances had the writers copied their opinions and practice from their predecessors. In most cases the writer appears to have been ignorant of what had been done before, and indeed, he has generally avowed a belief that his method was new and altogether original.

This fact may not be difficult of explanation when we call to mind the infrequency of dislocations of the hip, and that fifty years ago the number of surgical books, and especially of Medical Journals was very much less than at present: so that great and valuable truths often died with their discoverers, or were known and remembered only by a few. But it is possible that it has a deeper significance, and that it implies some defect in the procedure, or serious danger, in consequence of which it has from time to time lapsed into disuetude and finally complete oblivion.

It is for the purpose of aiding in the elucidation of this question; and that we might, if possible, determine what accidents or injuries are likely to accompany the reduction of dislocations of the hip by manipulation, that I have had these tables constructed, and with the following results:

The total number of cases is 64. Of 35 cases in which the age is stated the average is 24 years, the youngest being two years and one month, and the oldest 52 years. Of 51 cases stated, 45 were males and 6 females. Of 57 cases stated, 30 were dislocated upon the dorsum ilii; 14 into the ischiatic notch; 10 upon the foramen ovale, and 3 upon the pubes. Of 53 cases stated, 37 had occurred within 24 hours of the time at which the reduction by manipulation was attempted; 4 within ten days; 4 within three weeks; 1 at four weeks; 1 at five weeks; 1 at six weeks; 1 at seven weeks; 1 at twelve weeks; 2 at twenty-four weeks; and 1 at thirty weeks.

In 50 cases stated, pulleys had been previously trid and had failed in 8; Jarvis' adjuster in 1; Jarvis' adjuster and manipulation in 1; other modes of extension in 6. In 34 cases no means whatever had been previously employed.

Of 52 cases stated, ether was employed to diminish the resistance of the muscles, and to aid in the reduction by manipulation, in 16; chloroform in 13; chloroform and ether in 2; vena section in 3, with or without opium and antimony; morphine alone in 1; and no means whatever in 17 cases.

Of 41 cases stated, 28 were reduced in the first attempt; 7 in the second; 4 in the third, and 2 in the seventh.

In 7 examples the head of the femur has been thrown from one position to another upon the ilium, traveling from the dorsum to the foramen ovale and back, or from the ischiatic notch to the foramen ovale, or from the dorsum to the ischiatic notch, &c.: in some instances these changes being made as often as seven times in succession. In two of these examples the limb has been finally reduced by pulleys, and in one instance an abscess with morbus coxarius has ensued. In no other case has any permanent injury been known to follow these changes of position of the head of the bone.

In 1 case the thigh was broken during manipulation, through its lower third.

20 cases are known to have got well speedily, or within a few weeks after the reduction of the bone. 1, the case of compound dislocation, died in three weeks.

The following seem to be the only complete failures:

Case XXII, a New York Hospital case, of one month's standing, was finally reduced by pulleys.

Case XXIX. N. Y. Hospital case, of seven weeks' standing. Pulleys were finally applied and the neck of the femur broken, but no reduction accomplished.

Case XVIII. N. Y. Hospital case, of three months' standing. Efforts at reduction had been previously made and the edge of the acetabulum is believed to have been broken. The first attempt by manipulation was successful, but it soon became reluxated, and it could not again be reduced.

Case LXII. Femur was broken by manipulation. The account is incomplete.

Case LXI. Dr. Gunn's case. Recent. Simple manipulation failed. Manipulation with extension, successful.

Case LVII. N. Y. Hospital case. Existed only a few hours. Seven attempts with manipulation, failed. Second attempt by extension, successful.

Of these six failures two are known to have been recent cases, and three to have been ancient; but in only two of the six is a positive triumph of the pulleys over manipulation established: one a recent case, and one an ancient case; while on the other hand, eight were reduced by manipulation after pulleys had failed; two after Jarvis' adjuster had failed; three after manual extension had been tried; and three after extension, the precise character of which is not stated. In all sixteen triumphs of manipulation over extension.

It will be noticed that the great majority of the cases from which this analysis has been made, occurred in private practice, and it is unnecessary to say that such statistics do not furnish the most reliable basis for conclusions. As a general rule, unsuccessful cases are not published by private practitioners, but successful cases are pretty certain to be made known; while on the other hand, a series of cases furnished by any single hospital will generally be found to have given both unsuccessful and successful cases. The writer has heard lately of a complete failure to reduce by manipulation in a recent luxation of the hip, after repeated efforts on several successive days, and where skillful surgeons were in attendance; but it is believed that no account of the result has been published.

At least four of the unsuccessful examples recorded were hospital cases, and were all found in the report of 15 cases reported by Markoe, and Van Buren, from the New York Hospital! This is a startling fact, especially when we consider the skill of the several gentlemen who were the operators in these cases; and it plainly renders a new series of statistics necessary, drawn solely from the experience of one or more similar large establishments, before we shall be prepared to decide positively upon the relative value of the two procedures.

Note. Indeed the writer is in doubt whether he ought to regard cases 61 and 62 as examples of failure to reduce by manipulation, since in the first case the reduction was finally accomplished by manipulation *aided* only by manual extension, while in the second case, (62,) we are only informed that the femur was broken by manipulation; but we do not positively know that it was not reduced. If we are permitted to reject these two, then we have no reported cases of failure but the four New York Hospital cases, which occurred in a total of fifteen, and stand in the ratio of more than one failure in every four cases.

